# Genomic strategies for drug repurposing

**DOI:** 10.1186/s43046-024-00245-z

**Published:** 2024-11-11

**Authors:** Kirtan Dave, Dhaval Patel, Nischal Dave, Mukul Jain

**Affiliations:** 1 Department of Life Sciences, Parul Institute of Applied Sciences, Parul University, Vadodara, Gujarat India; 2 Bioinformatics Laboratory, Research & Development Cell, Parul University, Vadodara, Gujarat India; 3 Gujarat Biotechnology University, Gandhinagar, Gujarat India; 4 Cell & Developmental Biology Lab, Research and Development Cell, Parul University, Vadodara, Gujarat India

**Keywords:** Drug repurposing, Genomics, Proteomics, NGS, Cancer

## Abstract

Functional genomics, a multidisciplinary subject, investigates the functions of genes and their products in biological systems to better understand diseases and find new drugs. Drug repurposing is an economically efficient approach that entails discovering novel therapeutic applications for already-available medications. Genomics enables the identification of illness and therapeutic molecular characteristics and interactions, which in turn facilitates the process of drug repurposing. Techniques like gene expression profiling and Mendelian randomization are helpful in identifying possible medication candidates. Progress in computer science allows for the investigation and modeling of gene expression networks that involve large amounts of data. The amalgamation of data concerning DNA, RNA, and protein functions bears similarity to pharmacogenomics, a crucial aspect in crafting cancer therapeutics. Functional genomics in drug discovery, particularly for cancer, is still not thoroughly investigated, despite the existence of a significant amount of literature on the subject. Next-generation sequencing and proteomics present highly intriguing opportunities. Publicly available databases and mining techniques facilitate the development of cancer treatments based on functional genomics. Broadening the exploration and utilization of functional genomics holds significant potential for advancing drug discovery and repurposing, particularly within the realm of oncology.

## Introduction

### Functional genomics and drug repurposing

**Fig. 1:**
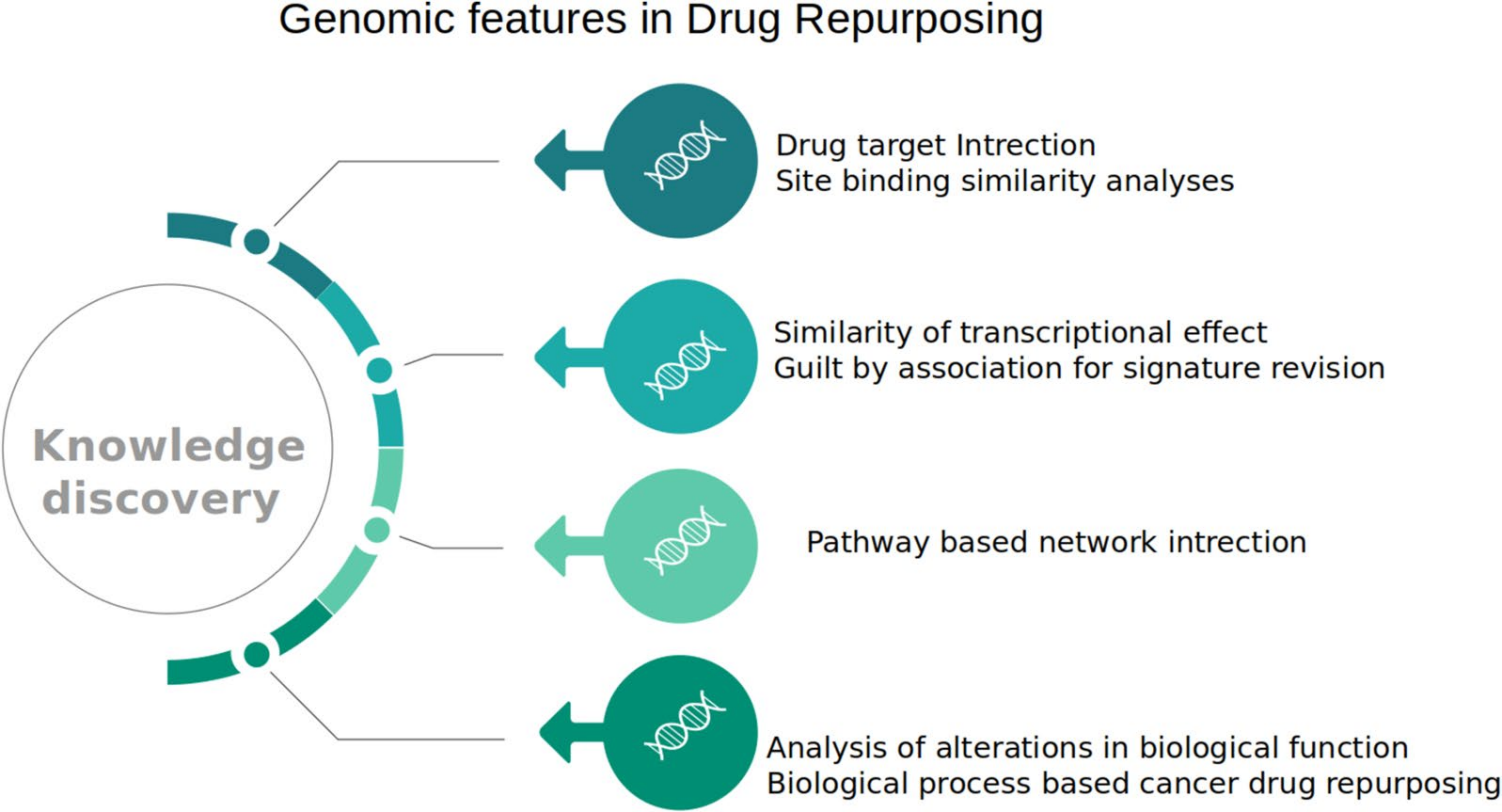
Overview of computational functional genomics techniques chosen for application in medication research

The field of functional genomics investigates the roles that genes and the products they produce play in biological systems, including tissues, cells, and organisms. Functional genomics can help identify the molecular mechanisms of diseases and potential targets for drug intervention [1]. Drug repurposing is the process of finding new therapeutic uses for existing drugs that have already been approved or tested for other indications [2]. Drug repurposing can save time and money compared to the traditional drug discovery process and can also provide novel treatments for medical needs [3]. Genomics plays an important role in drug repurposing, as it can provide insights into the molecular signatures of diseases and drugs and reveal the potential interactions and effects of drugs on different biological pathways and networks [4]. One of the common strategies for drug repurposing based on genomics is to compare the gene expression profiles of diseases and drugs and identify drugs that can reverse or modulate the disease signatures [5]. Another strategy is to use Mendelian randomization, which is a method that uses genetic variants as natural experiments to infer causal relationships between exposures and outcomes, such as drugs and diseases [4, 6]. Moreover, this field of research looks at the physiological, cellular, and/or biochemical properties of each gene product [7]. The aim is to determine the global effects of the genome on the phenotype. Thanks to these advancements, studying disease models and targeted medications has become a data-intensive field that can only be grasped by utilizing concurrent advances in computer science, such as knowledge bases, big data mining techniques, machine learning techniques, and artificial intelligence [8]. These techniques have been used in the research and development of cancer drugs. The technique entails integrating data from several facets of DNA sequencing, gene expression, and protein functionality, similar to pharmacogenomics. The processes involved in this context are transcription, which involves both coding and noncoding sections; protein translation; interactions between proteins and DNA/RNA; and protein–protein interactions. Afterward, they use this data to create interactive and dynamic networks that control gene expression, cell differentiation, and the advancement of the cell cycle [9]. Then they performed a methodical search on January 31, 2024, using the PubMed database (www.ncbi.nlm.nih.gov/pubmed) to collect pertinent material on these subjects. They specifically targeted the search query to uncover information on the functional aspects of genomics and cancer, particularly focusing on pharmacology and various omics fields like genomics, transcriptomics, proteomics, metabolomics, and interactomics. The search excluded any review articles. They obtained a total of 584 outcomes, with 68 specifically focusing on genetic association investigations of minor gene sets associated with illness characteristics. Figure 1 shows that they only looked into a few important methods, like using functional genomics to find new drugs and find new uses for old ones, especially in the context of cancer therapies. This way, they were not just talking about big discoveries that might lead to this result [10]. They utilize Next-generation sequencing (NGS) for cancer medication research purposes [11]. Additionally, they employ functional proteomics-based methodologies to identify cancer targets and facilitate knowledge discovery. Table 1 shows a complete list of all the public databases and data mining tools that are commonly used in the main ways that functional genomics-based approaches are being used to develop new cancer treatments. These scenarios will be briefly discussed and illustrated in the following.

**Table 1:** Examples of information sources and computer programs that are utilized in the data science approach to medication repurposing based on knowledge from functional genomics

Databases and resources for functional genomics		
AmiGO (search utility for GO)	Search tool for Gene Ontology (GO)	http://amigo.geneontology.org/
Gene Ontology (GO)	A standardized classification system for gene functions and products	www.geneontology.org/
HUGO Gene Nomenclature Committee	Assigns standardized nomenclature to human genes	www.genenames.org/
Database for Annotation, Visualization, and Integrated Discovery (DAVID)	Specifies tools for the annotation of functions and gene set enriched analysis	https://david.ncifcrf.gov
NCBI gene index database	National Center for Biotechnology Information’s database for gene information	www.ncbi.nlm.nih.gov/gene/
GeneCards	Comprehensive database for human genes, their products, and their associations	www.genecards.org
gnomAD	Genome aggregation database	https://gnomad.broadinstitute.org/
Human cell atlas	Identifying the individuals in the general population who can tolerate heterozygous or homozygous loss of function and relevant cell expression	https://www.humancellatlas.org/
LINCS	Gene expression perturbation	http://www.lincsproject.org/
CRISPR KO	The target responds with the appropriate perturbation(s)	https://depmap.org/portal/depmap/
	Gene-to-drug compatibility and examples Open Targets Platform	https://www.targetvalidation.org/
Human diseases		
Online Mendelian Inheritance in Man (OMIM) database	Catalogs human genes and genetic diseases, with a focus on the phenotypic-genetic relationships	www.ncbi.nlm.nih.gov/omim
MalaCards: The human disease database	Database providing comprehensive information on human diseases	www.malacards.org
DiseaseGenes database	A database cataloging genes associated with various diseases	www.jbldesign.com/jmogil/enter.html
The Human Disease Genetics Database (HPGDB)	Database focused on the genetics of human diseases	https://humandiseasegenetics.org/hpgdb/
Comparative Toxicogenomics Database (CTD)	Integrates toxicology and genomics to advance understanding of the effects of environmental exposures on human health	http://ctd.mdibl.org
Biological pathways		
Pathway Commons	A resource for biological pathway analysis and integration	www.pathwaycommons.org
Kyoto Encyclopedia of Genes and Genomes (KEGG)	Provides information on biological pathways, diseases, and drugs	www.genome.jp/kegg/
Drugs, small molecules, and/or (potential) targets		
DrugBank database	Database containing information on drugs, their targets, and mechanisms of action	www.drugbank.ca
Connectivity map (CMap)	Analyzes the effects of small molecules on gene expression and cellular phenotypes	https://clue.io/cmap
DrugSig	Identifies gene expression signatures related to drug response	http://biotechlab.fundan.edu.cn/database/drugsig/
Thomson Reuters Integrity database (non-free)	Database providing information on drugs and their properties	https://integrity.thomson-pharma.com
ChEMBL	Database including bioactive compounds having drug-like characteristics	www.ebi.ac.uk/chembl/
UniProtKB/Swiss-Prot	Comprehensive resource for protein information	www.uniprot.org
Gene Expression Omnibus (GEO)	Repository of high-throughput expression of genes and functional genomics datasets	www.ncbi.nlm.nih.gov/geo
Software		
R software	Programming language and software environment for statistical computing and graphics	http://CRAN.R-project.org/
Drug versus disease (DvD)	Analytical approach comparing drug and disease-related data	www.ebi.ac.uk/saezrodriguez/DVD/
GeneTrail	Software for the statistical evaluation of biological pathways	http://genetrail.bioinf.uni-sb.de/
Bioconductor	Open-source software for the analysis and comprehension of high-throughput genomic data	http://bioconductor.org/
R package ‘org.Hs.eg.db’	R package providing gene annotation for the human genome	https://bioconductor.org/packages/org.Hs.eg.db/
R package ‘GO.db’	R package providing Gene Ontology annotations	http://bioconductor.org/packages/GO.db/
R package ‘dbtORA’	R package for over-representation analysis in genomics	https://github.com/IME-TMP-FFM/dbtORA
Graphviz	Open-source graph visualization software	www.graphviz.org
Genome-wide CRISPR screen	Genetic screens to identify cancer dependencies	https://score.depmap.sanger.ac.uk
	The Dependency Map (DepMap) site aims to enable scientists to make discoveries about cancer vulnerabilities by offering free access to major cancer dependencies, analytical, and visualization tools	https://depmap.org/portal/
	BioGRID Open Repository of CRISPR Screens (ORCS)	https://orcs.thebiogrid.org/
Literature		
PubMed	A comprehensive database of biomedical literature provides access to a vast collection of articles, research papers, and other scholarly materials	www.ncbi.nlm.nih.gov/pubmed/
Scopus		https://www.elsevier.com/en-in/products/scopus
Web of Science		https://clarivate.com/products/scientific-and-academic-research/research-discovery-and-workflow-solutions/webofscience-platform/
Google Scholar		https://scholar.google.com/

Functional genomics, computational drug development, and job reassignment have all derived advantages from the various applications of genetic research [12]. Additionally, computational molecular docking methods try to find the best places for small molecules to bind by looking at how well the chemical structures match up with each other [13]. This is used for protein-level target identification and repurposing in drug discovery, depending on the compound's uniqueness in a pharmacological setting [14]. If you possess a known medicine that interacts with several target structures and proteins, you can perform virtual screens in conjunction with protein–protein interaction research. Alternatively, you can dock a protein–protein interaction from a database onto the protein of interest. Only research projects that use nuclear magnetic resonance, crystallography, or comparative modeling to create the three-dimensional structure of a target can use molecular docking to get accurate results [15]. A sophisticated network of phosphorylation signaling facilitates the functioning of several cell types [10–12]. This network contains the MAP2K kinase (MAP2K/MKK), the MAP3K kinase (MAP3K/MEKK), the MAP3K kinase (MAP4K), and MAPK. The MAPK signaling pathway is initiated by pattern recognition receptors (PRRs) and pathogen-associated molecular patterns (PAMPs), leading to the phosphorylation of additional components in the downstream pathway. Therefore, this stimulation gets both adaptive and innate immune responses. The MAP kinase family involves a protein known as MAPK1, also known as ERK2 (extracellular signal-regulated kinase 2). It is crucial for various biological processes, such as cell growth, specialization, and viability. Researchers have identified MAPK1 as a crucial element in various disorders, including cancer. In a study on the protein MAPK1, scientists used computational molecular docking to successfully add drugs from the DrugBank database [16, 17] to the 35 crystal structures of MAPK1 [18]. Furthermore, the MAPK cascade consists of a minimum of three-tier conserved protein kinases, specifically MAP3Ks, MAP2Ks, and MAPKs [19, 20]. The PPI network’s prediction revealed a deductive signaling pathway for the genes involved in the Eg-MAPK cascade. This knowledge is useful for conducting further studies on these genes’ roles [21]. Celecoxib, which blocks the enzyme cyclooxygenase-2 (COX-2), seems to have an effect on the transmembrane protein cadherin-11, which has been linked to the development of rheumatoid arthritis. Researchers utilize transcriptional responses to alter the patterns of gene expression in response to medication treatment as compared to untreated samples [22]. In addition, scientists have reutilized aspirin, a widely recognized analgesic, for the purpose of averting cancer and cardiovascular illness [23, 24]. Comparing different gene expression patterns, like those found in GEO (Gene Expression Omnibus), is the starting point for further steps [25], which can be caused by medicine or by illness. They can gain knowledge of medication-induced changes in gene expression by comparing the expression profile of a cell before and after drug exposure. Understanding the alterations in gene expression associated with the condition of interest is crucial. More precisely, a phenomenon arises when a certain situation or therapy modifies the gene expression pattern of a cell or tissue, leading it to return to its initial state. This technique involves identifying genes or proteins that are associated with a specific biological process or function. They accomplish this by analyzing their patterns of co-expression or co-occurrence with known genes or proteins already associated with that process. Instead of focusing on specific pharmacological targets or theories of their functioning [26], phenotypic drug discovery approaches aim to tackle the complex characteristics of diseases that remain poorly understood [27]. Molecular drug targets act as a connection between medication and disease-related biological processes. Scientists hypothesize that changes in the function of one or many biological systems implicated in a disease’s pathophysiology are responsible for its development. Listed biological processes that are included in the knowledge base of GeneOntology (GO) [28].

### GWAS and drug repurposing

**Fig. 2:**
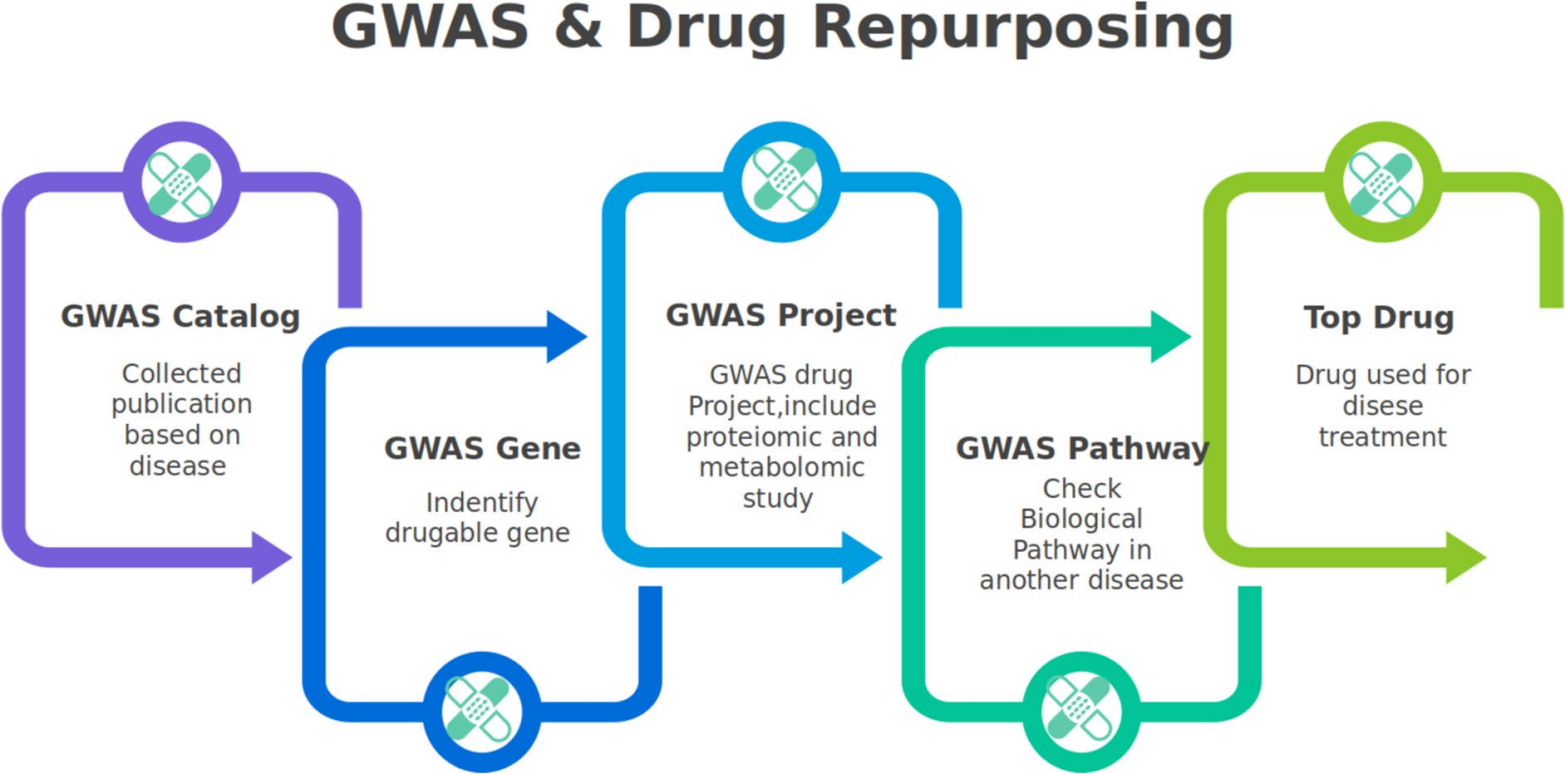
Flowchart of GWAS and drug repurposing

Genome-wide Association Studies (GWAS) sent out molecular sails to cruise the genomic seas in their vast hunt for new therapeutic routes [29]. This research carefully searches the wide breadth of our genomic landscape and the invention of cryptic variations that are closely linked to a variety of disorders [30]. This genetic treasure map is the initial stage in the delicate dance of drug discovery and repurposing, since these variations serve as beacons, pointing researchers to previously unexplored lands of prospective therapies [31]. Thus, genome-wide association studies (GWAS) play an important role in medication repurposing by finding genetic variations related to the syndrome and contributing insights into prospective therapeutic targets [32]. Here are two cases that demonstrate the impact of GWAS in the drug repurposing process [33]. An unbiased method of genome-wide association studies (GWAS) is used to find correlations between genotype and phenotype in genotyping arrays or sequencing data [34]. GWAS has been carried out for thousands of features, improving our knowledge of human genetics and advancing the creation of treatments and diagnostics [35]. Genotype–phenotype association analysis (GWAS) has become a critical method for determining how genotype shapes phenotype due to the increasing quantity of genetic information [36]. investigations of genome-wide associations [37]. In recent years, there has been a notable increase in the incidence of genome-wide association studies (GWAS). This is due to breakthroughs in genotyping technology, the completion of the Human Genome Project, and the concomitant fall in genotyping costs. The primary objective of GWAS is to identify genetic variants linked to common diseases so that they can understand the biology of that disease. The information acquired might also assist in identifying new targets [38]. Some of these targets may apply to both GWAS-studied disease phenotypes and drug-treated disorders. This might result in narcotics being transported to new locations [39]. The pharmaceutical industry’s targets were 2.7 times more enriched in the GWAS gene set [40]. Sanseau and colleagues [41] modified the list of published GWAS features from the US National Human Genome Research Institute (NHGRI) [41]. Scientists found that genes linked to a particular disease trait had a higher likelihood of encoding proteins than genes found in other regions of the genome. These proteins could be utilized to produce novel therapeutic targets or develop pharmaceutical interventions to treat a particular ailment. In addition, they also found 92 genes whose GWAS phenotype was different from the prescribed course of therapy. This means that treatments targeting the products of these 92 genes may be tested for a new illness indication. Grover and colleagues 45 did a different study that used bioinformatics to look for possible repositioning opportunities [42]. They accomplished this by aligning gene targets identified for coronary artery disease with drug-related data amalgamated from three distinct drug-target databases(DrugBank) [43], Therapeutic Target Database, and PharmGKB [44]. GWAS data can be difficult to use for medication repositioning, and it is currently uncertain how useful it is. It might be hard to find the causal gene and/or gene variants when GWAS signals are present in areas with a lot of genes and a lot of linkage disequilibrium [39]. A different problem is a lack of clarity about the gene variant's direction of action; functional research must be conducted to ascertain this before deciding whether an activator or a suppressor is required to control the condition. When determining repurposing targets, it is best to utilize GWAS data intuitively because they do not give specific pathophysiological information [45]. It should be noted that there may be many more unique genes discovered, and our current understanding of the human genome is not complete. Genome-wide association studies (GWAS) are a powerful tool in the intricate game of genetic discovery [33, 46]. They meticulously navigate the vast expanse of our genetic blueprint to unveil the intricacies inscribed within our DNA strands. By adeptly mapping the terrain of diseases and pinpointing elusive variants that may harbor the key to crafting personalized treatment regimens, these endeavors serve as genetic cartographers. As the genetic constitution are deciphered, genome-wide association studies (GWAS) unveil a startling revelation by instructive distinct cohorts of patients with individual molecular narratives. Patient decomposition empowers the construction of homogeneous cohorts tailored for personalized, precision therapies, epitomizing the art of distilling precision from complication. This way is also heralded as an alchemist in drug development, unlocking gateways into the enigmatic realms of genes and pathways involved in disease progression. These inspections imply the presence of potential drug targets awaiting the finding or utilization of ancient manuscripts. Acting as connections, genome-wide association studies (GWAS) intricate genetic variants associated with syndromes to currently available medications, thereby facilitating access to therapeutic interventions suited to specific genetic subgroups. The relation between pharmacology and genetics is focused and the rapid progression of drug development. Genomic understanding gives rise to precision medicine, leveraging an individual’s distinct genetic makeup to formulate personalized treatment strategies. Each genetic variation in the symphony dictates the response to a particular medication, culminating in a customized and efficacious regimen. In clinical trials, GWAS serves as a guiding light, directing attention toward patient populations most likely to benefit from specific medications. Through this targeted approach, clinical trials evolve into meticulously orchestrated endeavors where efficacy and efficiency synergistically prevail. These genetic discoveries transform into viable drug targets, allowing for the creation of new drugs as well as the repurposing of already approved ones. The figure shows the flow process (Fig. 2).

So, incorporating drug repurposing into this genome-wide association studies (GWAS) sharpens our focus on the intricate genetic syndromes. By unraveling specific genetic alterations highlighted by GWAS, researchers gain insights essential for drug repurposing. Using information about disease subtypes, researchers can scour the vast field of pharmacology for previously developed drugs that could potentially target specific disease sub-types identified by GWAS. In the realm of precision medicine, this path transcends conventional treatment paradigms. Repurposed drugs, tailored to specific disease subtypes identified through genome-wide association studies (GWAS), align with the principles of precision medicine. This program regards the unique genetic makeup of individual patients or specific subgroups, offering a nuanced and effective approach to treatment. Validation is based on clinical data from repurposing trials, which shows that certain drugs are effective at treating certain types of diseases. This process shows and validates disease subtypes based on genetic markers, and gives confidence in the precision medicine path. Moreover, medication repositioning has been shown an effective accelerator for speeding up the therapeutic development process. By bypassing the need to start from scratch, researchers capitalize on the existing safety profiles and pharmacological data of repurposed drugs. This accelerates the translation of genetic insights into actionable treatment options for specific disorder subtypes, paving the way for more efficient and targeted interference. The genome-wide association studies (GWAS) guided disease subtype mapping and drug repurposing heralds a new era of personalized treatment strategies. Patients within a particular disease subtype stand to benefit from treatments finely tailored to their specific genetic vulnerabilities, promising improved therapeutic outcomes. The combination of genome-wide association studies (GWAS) and drug repurposing is a big step towards a future where complex genomic landscapes guide therapeutic efforts. This could lead to a more accurate, effective, and re-source-efficient healthcare system or rediscovery, laying the foundation for more streamlined drug development pipelines.

Drug repurposing by single nucleotide polymorphism (SNP) research is an optimistic method for revealing new indications for current medications. Some researchers have used genomic analysis and functional annotations to discover potential medication repurposing candidates for a variety of disorders, including metabolic disorders. To generate novel theories for cancer and uncommon diseases [47], computational approaches have evolved that incorporate many forms of chemical and genomic data, including chemical structures, drug-target interactions, pathways, and disease-gene associations [48]. In addition, researchers can repurpose similar drugs for new uses by applying SNP analysis and genetic data. For instance, a probe on depression found genes like IL6R that are strongly linked to depression and employed 5885 SNP analysis to find drug applicants for repurposing. The outcome of this study included the identification of potential medications like sarilumab and satralizumab [49]. Assuming the impact of drug target disruption, scientists have used SNP analysis to get new repurposing possibilities for identifying promising therapeutic classes for clinical trials.

### Gene expression signatures and drug repurposing

Gene expression characteristics can be exploited in medication repurposing to reveal new therapeutic using medicines that are already on the market. Potential therapeutic can be found for repurposing by evaluating the overall transcriptome effects of medications and comparing them to gene expression features [50]. Drug repurposing for many forms of cancer has taken up the concept of gene regulation [51], which includes many drugs that can restore gene expression variations caused by a disease or syndrome to a base level. A computational system based on gene expression signatures and connection scores has been created to find possible repurposed medicines. These methods have demonstrated potential for locating therapeutic for conditions like low-survival malignancies and Psoriasis vulgaris. Overall, gene expression plays an important role in the area of drug repositioning because it provides information about the molecular effects of medicines and their potential therapeutic uses [52].

Genomic research unravels the dynamic interactions of gene expression patterns within biological systems [53]. As treatments or environmental stimuli affect modifications in the transcriptome, the study of signature regression [54] deals with the phenomenon of whether the gene expression profile returns to its original state over time [55]. This process is translating the long-term impacts of therapeutic interactions [56], guiding researchers to notice the adaptability of cellular responses. The research on transcriptome signature regression holds immense significance, particularly in drug development and precise medicine. It offers an understanding of how biological systems respond to external influences [57] and whether the internal changes are reversible, providing critical insights [58] for changing interactions and improving treatment strategies. In unveiling the dynamics of gene regulation, transcriptome signature reversion emerges as a vital concept shaping the landscape of genomic exploration, promising advancements in our comprehension of cellular behavior and potential avenues for therapeutic discovery [59]. The main advantage of genetic markers in oncology-based medication repurposing for various forms of cancer is that they have been widely employed to discover novel applications for previous treatments.

The standard protocol for a transcriptome signature reversion (TSR) drug repurposing study typically involves employing differential gene expression analysis to elucidate the specific genes that exhibit altered expression patterns in tumor tissue compared to adjacent healthy normal tissue. This foundational step is important for identifying potential therapeutic candidates and understanding the molecular underpinning of the syndrome. By pinpointing genes that are upregulated or downregulated in the tumor, researchers gain valuable insights into the unique transcriptomic signature associated with the pathological state. This differential gene expression analysis serves as the basis for subsequent investigations, guiding the selection of candidate drugs for repurposing. The path not only shows the molecular landscape of the syndrome but also provides a rational framework for searching existing drugs with the potential to modulate the identified gene expression changes, thereby advancing drug-repositioning efforts in the realm of cancer therapeutics. Mainly, the Connectivity Map (CMap) database and the Library of Integrated Network-Based Cellular Signatures (LINCS) L1000 database are two well-known tools for mapping changes in gene expression patterns that are seen in the disorder. Moreover, many studies using this technique have resulted in medications with anti-cancer activity against specific tumor types, showing that TSR has a strong prediction power for selecting candidate medicines for repositioning. The idea of TSR in cancer has the best-known systematic evidence. Research found that the chemicals evaluated in a single cell line of that tumor type had a median half-maximum inhibitory concentration (IC50) that may be utilized to reverse the gene expression profiles of breast, liver, and colorectal cancers. Nonetheless, rather than concentrating on the upstream “cause” of the proliferative phenotype that defines a certain cancer type, TSR-identified drugs may instead target the downstream proliferative. It has been demonstrated that medications that reduce cell viability have comparable disruption patterns of gene expression, which are connected to transcription factors that regulate cell death, proliferation, and division time. To put it another way, a bigger projected inversion of a cancer gene expression profile based on the pharmacological signature may indicate the treatment’s general success in limiting cell proliferation rather than its specificity to the kind of tumor under investigation. As a result, TSR as it is now used is not as valuable for medication repurposing as previously thought since it is insufficiently precise to identify which treatments are most likely to be successful against a certain form of cancer [53]. To increase TSR accuracy, medication signatures indicating downstream gene expression effects resulting in fewer cells surviving may be eliminated.

One of the case studies on Dexamethasone resistance, which is a bottleneck in the treatment of acute lymphoid leukemia (ALL), was discovered to be reversed when treated with sirolimus. Gene signatures of dexamethasone resistance and sensitivity in both healthy and ill individuals were generated from bone marrow leukemic cells and utilized as query signatures in CMap. The query fingerprints for dexamethasone sensitivity showed a strong connection with sirolimus (a mTOR inhibitor). Furthermore, Gene Set Enrichment Analysis (GSEA) [60] demonstrated a strong association between down-regulated genes in sirolimus-treated lymphoid cells and up-regulated genes in glucocorticoid-resistant cells. In vitro investigation employing the lymphoid cell line CEM-c1 demonstrated that pre-treatment with 10 nM sirolimus improved dexamethasone sensitivity in both dexamethasone-resistant and non-resistant ALL cells. Based on encouraging computational and in vitro results, a proof-of-concept was developed to justify the present position of sirolimus in phase I studies with dexamethasone for relapsed ALL (NCT01403415) [61]. Another drug fluorouracil (5-fluorouracil or 5-FU) is an anti-metabolite medication used primarily in the treatment of cancer. Its mechanism of action involves interfering with the normal function of nucleic acids (DNA and RNA) and disrupting the processes necessary for cell proliferation. Fluorouracil’s mechanism of action involves multiple interconnected processes that ultimately lead to inhibition of DNA and RNA synthesis, disruption of nucleic acid function, and induction of apoptosis in rapidly dividing cells, such as cancer cells [62]. This multimodal mechanism accounts for its efficacy as a chemotherapeutic drug in the treatment of numerous malignancies, including colorectal, breast, and skin cancers. High dosage 5-FU/paclitaxel combined with doxorubicin [63] was shown to be effective in the treatment of breast cancer. Methotrexate, a folate/folic acid antagonist, binds to the dihydrofolate reductase (DHFR) enzyme and suppresses the synthesis of DNA and RNA building blocks. DHFR converts folic acid to dihydrofolate, which is subsequently reduced to tetrahydrofolate (THDF) for use by thymidine synthetase. Methotrexate binding to DHFR reduces purine and pyrimidine production in cells, hence inhibiting cell cycle progression in the S phase. Methotrexate came into existence in the 1940s as a replacement for folic acid after clinics reported cases of reduced leukemic cell count (acute lymphoblastic leukemia) caused by dietary folic acid shortage (Table 2) [64, 65].

**Table 2:** List of drugs repurposed for cancer target

Drug	Discovered	Repurposed	Ref
Anastrazole	Ovulation induction	Breast cancer	[66]
Capecitabine	Colon cancer		
Cyclophosphamide	As immuno-modulator in autoimmune		
Everolimus (Votubia, Evertor)	Immunosuppressants during organ		
Exemestane	Ovulation induction		
Fluorouracil	Keratoacanthomas, actinic kerato-		
Fulvestrant	Anti-estrogen		
Gemcitabine	Anti-viral drug		
Goserelin	Prostate cancer, uterine fibroids, precursor cleavage		
Methotrexate	Leukemia		
Paclitaxel	Ovarian cancer, atrial restenosis		
Raloxifene	Osteoporosis in postmenopausal		
Thiotepa	Immunosuppressant		
Letrozole	Ovulation induction		
Toremifene	Infertility with an ovulatory disorder		
Vinblastine	Hodgkin lymphoma, non-Hod-		
larithromycin, pioglitazone,	Antibiotic	Non-small cell lung cancer	[67]
Digoxin	Treatment for cardiac diseases	Anti-cancer	
Disulfiram (Antabuse)	Reduces ethanol tolerance in alco-	Metastatic breast cancer and Alzheimer’s	[68] [20] [69] [70]
Mibefradil (Posicor)	Anti-hypertensive, calcium channel	Short-term use as an adjuvant in cancer	
Losartan	Blood pressure reduction	Alzheimer disease	
Nelfinavir	HIV protease inhibitor	Solid tumors	
Metformin	Diabetes	Anti-nonsmall cell lung cancer, and aug-cancer	
Itraconazole	Anti-fungal	Anti-cancer	[71]
Mebendazole	Anti-parasitic/helminthiasis/anti-infective	Brain cancer (i.e., medulloblastoma and	
Mycophenolic acid	Immunosuppressant	Anti-cancer	

**Fig. 3:**
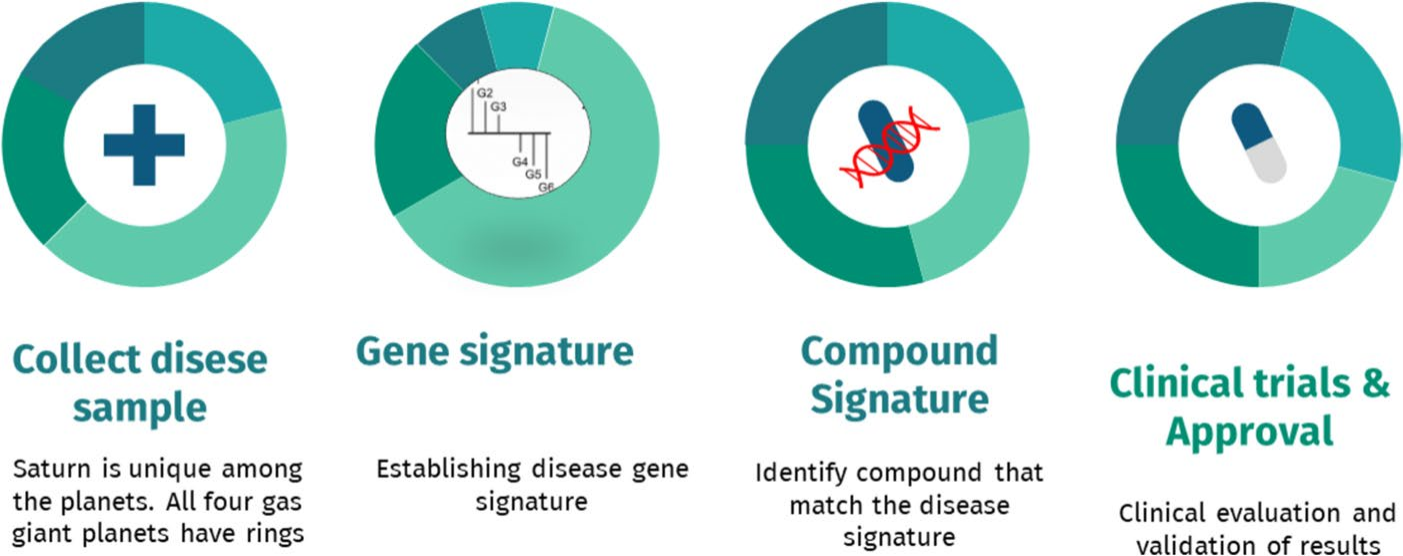
Stages of gene expression signatures and drug repurposing

Another study found that by utilizing publically accessible molecular data on gene expression in inflammatory bowel disease (IBD) samples, the scientists examined a database of small-molecule therapeutic candidates. Gene expression signatures were matched across a variety of known therapeutic compounds to a gene expression signature for IBD from the National Centre for Biotechnology Information’s Gene Expression Omnibus (GEO) [72]. GEO is a worldwide public database that stores microarrays, next-generation sequencing, and other types of high-throughput functional genomics data. Generated predictions for drug-illness associations are based on the idea that if a medicine has a gene expression profile that is opposed to the disease expression profile, it signals a viable therapy for that condition. The separate investigated model predicts that the corticosteroid medicine prednisolone would have the opposite gene expression profile as Crohn’s disease; prednisolone is a well-known therapy for Crohn’s disease symptoms [73]. When examined on the gene expression profile for ulcerative colitis, the medication topiramate showed the reverse result [74]. This implies that it could potentially serve as a therapeutic agent. Topiramate, a safe and FDA-approved medicine for treating epilepsy and migraine headaches, presents a viable option for repurposing to treat IBD. A study that looked back at administrative claims found no evidence to support this suggestion of topiramate as an alternative strategy. If two drugs have the same effect on gene expression, they may have the same therapeutic use, even if they do not have the same chemical structure or direct target. The idea is that discovering another medicine with a similar genetic mechanism of action might lead to a new therapeutic with greater efficacy or safety. Many researchers created an automated technique that uses similarity in transcriptional response in human cell lines following drug treatment (across various cell lines and doses) to predict similarities in medication impact and mechanism of action. The result is a publicly available Mode of Action by Network Analysis (MANTRA; http://mantra.tigem.it/) tool for analyzing new medications' modes of action and identifying candidates for drug repositioning [73] (Fig. 3).

An approach called expression quantitative trait loci analysis, which combines genomics and transcriptomics, has also been useful for connecting genetic variations with gene expression that is specific to a tissue. Together, these tools, in combination with in vitro reporter assays and functional in vivo assays, can help identify genes associated with disease for further exploration. They should continue to employ and improve this strategy, as combining various omics to generate more holistic hypotheses and approaches represents an important advancement in the use of existing data. Similarly, comparing the expression profile before and after drug exposure can elucidate the changes brought about by drugs on the transcriptional program. Based on transcriptional data, several computational approaches propose drug repurposing. The differential gene expression signature reflects the impact of a drug on gene expression. They can compare drug-induced transcriptional changes with disease-associated gene expression. If there is a negative correlation between the disease-induced transcriptional changes and the drug-induced transcriptional changes, then the drug may have efficacy in treating the disease. The drug may be able to reverse the disease-induced gene expression and mitigate the disease phenotype [72].

## Conclusion

Drug repurposing is undergoing a revolution thanks to functional genomics, genome-wide association studies (GWAS), and gene expression profiles, opening up new medicinal discovery paths. Functional genomics examines the roles of genes and their products in biological systems. Understanding the molecular causes of diseases, identifying drug targets, and offering a more cost-effective approach to drug discovery are valuable contributions to this cancer research. GWAS is a study of genetic variants associated with diseases, serving as a genetic roadmap that directs researchers toward potential therapeutic candidates and allows personalized medicine by categorizing patients. Targeted treatments have become possible because of the increasing recognition of the complex link between genetic makeup and personalized medicine. Some issues still exist, though, including the problem of disequilibrium and the demand for DNA functional evaluation. Like the intriguing notion of transcriptome signature reversion (TSR), these signatures offer a valuable tool for scientists to uncover novel applications for existing drugs. Researchers can gain insights into potential therapeutic uses by comparing the alterations in gene expression patterns caused by drugs with disease-specific patterns. TSR-based techniques exhibit potential, but further investigation is needed to improve their accuracy of them in cancer. To overcome the obstacles and completely realize the potential of drug repurposing, collaborative work and advances in technology are crucial. In summary, drug repurposing has the potential to accelerate cancer drug development, provide personalized treatments, and cut costs, ultimately leading to a more efficient and focused approach to therapeutic discovery.

## Data Availability

All data that support the findings of this study are available within the article.

## Abbreviations

GEO: Gene Expression Omnibus
GWAS: Genome-wide association study
NHGRI: National Human Genome Research Institute
GO: Gene Ontology
DEG: Differentially Expressed Genes
TSR: Transcriptome Signature Reversion
